# Endovascular Stent Placement of Juxtaanastomotic Stenosis in Native Arteriovenous Fistula After Unsuccessful Balloon Angioplasty

**DOI:** 10.5812/iranjradiol.11386

**Published:** 2013-08-30

**Authors:** Burak Özkan, Durmus Güngör, Utku Mahir Yıldırım, Ali Harman, Özgur Özen, Cüneyt Aytekin

**Affiliations:** 1 Department of Interventional Radiology, Baskent University School Of Medicine, Ankara, Turkey

**Keywords:** Endovascular Procedures, Vascular Fistula, Angioplasty

## Abstract

**Background:**

In hemodialysis patients, the most common problem in arteriovenous fistulas, as the best functional vascular access, is the juxtaanastomotic located lesions. Percutaneous transluminal angioplasty is accepted as the treatment method for juxtanastomotic lesions.

**Objectives:**

To assess juxtaanastomotic stent placement after insufficient balloon angioplasty in the treatment of autogenous radiocephalic or brachiocephalic fistula dysfunction.

**Patients and Methods:**

Between July 2003 and June 2010, 20 hemodialysis patients with autogenous radiocephalic or brachiocephalic fistula dysfunction underwent stent placement for the lesion located at the juxtaanastomotic region. Indications for stent placement were insufficient balloon dilatation, early recurring stenosis, chronic organizing thrombus and vessel rupture. The Kaplan-Meier method was used to calculate the stent patency rates. All patients who had fistula dysfunction (thrombosis of hemodialysis access, difficult access cannulation, extremity pain due to thrombosis or decreased arterial access blood flow) were evaluated by color Doppler ultrasound. The stenoses were initially dilated with standard noncompliant balloons (3 to 10-mm in diameter). Dilatation was followed by high pressure (Blue Max, Boston Scientific) or cutting balloons (Boston Scientific), if the standard balloon failed to dilate the stenotic segment.

**Results:**

Twenty-one stents were applied. The anatomical and clinical success rate was 100%. Seventeen additional interventions were done for 11 (55%) patients due to stent thrombosis or stenosis during follow-up. Our 1- and 2-year secondary patency rates were 76.2% and 65.5%, respectively and were comparable to those after balloon angioplasty and surgical shunt revision.

**Conclusion:**

Metallic stent placement is a safe and effective procedure for salvage of native hemodialysis fistula after unsuccessful balloon angioplasty.

## 1. Background

Native arteriovenous (AV) fistula is the best functional vascular access in hemodialysis patients (1). Specifically, a distal fistula has several advantages over a proximal one such as preservation of the remaining vascular channels for future use and a lower risk of steal syndrome. However, distal fistulas have a higher risk for stenosis and/or thrombosis. Juxtaanastomotic lesion is the main reason for inadequate shunt function in such fistulas (2). Percutaneous transluminal balloon angioplasty (PTA) is the primary endovascular technique in the treatment of hemodialysis access-related stenosis. Juxtaanastomotic lesions are known to be difficult to treat with this technique, because the etiology of these lesions is different from that of other outflow tract lesions. The high technical success of PTA in both dysfunctional polytetrafluoroethylene grafts and native arteriovenous fistulas has been reported. Early failure of the access after simple balloon dilatation may occur in some of the patients in whom metallic stent placement is considered mandatory for nonsurgical maintenance of the access (3, 4).

## 2. Objectives

In this article, we want to report our experience regarding metallic stent placement in treating peripheral outflow lesions in 20 AV fistula patients after insufficient balloon dilatation.

## 3. Patients and Methods

### 3.1. Patients

This is a retrospective and observational study. From July 2003 to June 2010, 20 hemodialysis patients with autogenous radiocephalic or brachiocephalic fistula dysfunction underwent stent placement for the lesion located at the juxtaanastomotic region (the area extending from 3 cm before to 3 cm after the anastomosis). Eighteen patients had radiocephalic (15 left, 3 right) and two had brachiocephalic (1 right,1 left) anastomosis. Three patients (15%) underwent percutaneous transluminal angioplasty for the same access previously (Table 1). All the patients had fistula dysfunction (thrombosis of hemodialysis access, difficult access cannulation, extremity pain due to ischemic arterial stenosis and thrombosis or decreased arterial access blood flow) and were evaluated by color Doppler ultrasound. 

### 3.2. Procedure

Thrombolysis was performed in twelve cases using 1-3 mg t-PA or 100.000-150.000 IU of urokinase 2-4 hours before the procedure. Urokinase or t-PA was diluted with 50cc saline and continuously injected with an automatic pump (with 25cc/hour speed). We used the 4 Fr micropuncture set (Cook Medical Inc, Bloomington, Indiana, USA) as a thrombolysis catheter. We placed the micropuncture set under the guidance of ultrasound, the entry point was the patent segment of the venous side reaching to the proximal side of the venous thrombus. If it was necessary, we added extraholes on the micropuncture set's vascular sheath to treat thrombus more efficiently. In addition, we added another micropuncture set to treat long segment thrombosis. For the residual thrombosis, balloon maceration was preceded in these cases. Additionally, Arrow-Treratola percutaneous thrombolytic device (Arrow International Inc, Pennsylvania, USA) was used after thrombolysis for mechanical thrombectomy in some cases. Diagnostic angiography was directly performed on eight patients who had efferent venous stenosis.

**Table 1.** Demographic Characteristics of the Patients

	Age	Gender	Fistula Localization	Previous İntervention
**Case 1**	48	Male	Left RS	
**Case 2**	53	Male	Left RS	
**Case 3**	14	Male	Left RS	
**Case 4**	55	Male	Left RS	
**Case 5**	38	Female	Left RS	
**Case 6**	43	Male	Right RS	
**Case 7**	46	Male	Left RS	
**Case 8**	41	Female	Left RS	
**Case 9**	66	Male	Left RS	PTA 5 months ago
**Case 10**	58	Male	Left RS	
**Case 11**	52	Male	Right RS	PTA 60 months ago
**Case 12**	75	Male	Left RS	
**Case 13**	73	Male	LEFT RS	
**Case 14**	59	Male	Left RS	
**Case 15**	73	Male	Left BS	PTA 16 months ago
**Case 16**	59	Male	Right BS	
**Case 17**	58	Male	Left RS	
**Case 18**	34	Female	Right RS	
**Case 19**	51	Male	Left RS	
**Case 20**	66	Female	Left RS	

Abbreviations: PTA, Percutaneous Transluminal Angioplasty; RS, Radiocephalic; BS, Brachiocephalic

All procedures were carried out under local anesthesia only. In some cases, intravenous midazolam was added. For all patients, the transvenous approach was tried for the first time. All punctures were done under the guidance of ultrasonography. An antegrade arterial approachusinga 4 Fr micropuncture set (Cook Medical Inc, Bloomington, Indiana, USA) from the cubital area was chosen if the venous side was completely occluded and when intervention failed to cross the lesion from the venous side. The “criss-cross” technique was used for the multiple stenotic segments to gain the access patency. Fluoroscopy and/or ultrasound-guided 5-7 Fr vascular sheath was placed close to the venous side of the anastomotic stenosis and after injection of the contrast material through the catheter or vascular sheath, the grade of the stenosis was obtained. The stenotic segment was tried to pass by 5 Fr catheters (Kump, Cook Medical Inc, Bloomington, Indiana, USA) with the 0.035 inch hydrophilic guide-wire (Terumo, Tokyo, Japan). The 2.8 Fr microcatheter (Fast Tracker infusion catheter, Boston Scientific, Massachusetts, USA) was used for the high grade stenotic segments with the help of 0.016 inch guide-wire (GT Radiofocus glide-wire, Terumo, Tokyo, Japan). The patients with stenosis at the juxtaanastomotic area, the side 3 cm closer to the anastomosis were included in the study. Stenoses were initially dilated with standard noncompliant balloons (3 to 10-mm in diameter). Dilatation was followed by high pressure (Boston Scientific Corporation, Boston, USA) or cutting balloons (Boston Scientific Corporation, Boston, USA), if the standard balloon failed to dilate the stenotic segment. After obtaining the angiogram, if the residual vessel stenosis was higher than 50%, the stent placement was the treatment of choice. Indications for stent placement were insufficient balloon dilatation, early recurring stenosis, chronic organizing thrombus and vessel rupture. Considering high-pressure or cutting balloon usage, the situation of a restenosis higher than 50% or inadequate blood flow for the dialysis treatment was termed as insufficient balloon usage. Early recurrent stenosis was named if stenosis occurred up to three months after balloon dilatation.

The initial criteria for choosing stent size was considering the whole segment of the stenosis and the stents varied from 8 to 80 mm in length and 4 to 10 mm in diameter. Based on the stenotic segments and their length, balloon expandable, self-expandable nitinol type stents were selected. In anastomotic sites, if there was a distinct discordance between the diameter of the arterial and venous size, the self-expandable stent that was suitable for the venous side was chosen.

### 3.3. Definitions and Outcome Measurements

In accordance with the definitions laid down by the Society of Interventional Radiology reporting standards, (a) primary patency rate is defined as the time between the initial intervention and the following repeat intervention (b) secondary patency rate is defined as the time of patency from the initial intervention until the access was surgically revised or abandoned or until transplantation, death,or loss to follow-up; (c) anatomic success is defined as less than 30% residual diameter in stenosis after therapy; (d) clinical success after treatment of a stenosis is defined as improvement of clinical and hemodynamic parameters with resumption of at least one dialysis treatment and (e) major complications include those that require therapy or hospitalization, cause permanent adverse sequelae, or death. Minor complications are those that require no or minimal therapy and have no consequences (5).

All procedures were carried out under local anesthesia only. In some cases, intravenous midazolam were added. Throughout the procedures, all patients were monitored with electrocardiography and by obtaining blood pressure, pulse rate, and capillary oxygen saturation measurements.

Informed consent was obtained from each patient.

### 3.4. Statistical Analysis

The Kaplan-Meier method was used to calculate the stent patency rates. All analyses were performed with SPSS for Windows Version 17 (SPSS Inc., Chicago, Illinois, USA). The study was approved by the local ethics committee of the research hospital. 

## 4. Results

Sixteen of the patients were male and four were female with a mean age,of 53.1 (14-75) years. Twenty-one stents were successfully placed at the juxtaanastomotic side and no major complication occurred during the procedure. The reason for the AV fistula intervention was occlusion in twelve patients (60%) and stenosis in eight patients (40%). These twelve cases had a history of percutaneous thrombolysis and/or thrombectomy. The transarterial approach was used in four patients (20%) and the transvenous in sixteen patients (80%). The indications for stent placement was chronic organizing thrombus in two patients (10%), early recurring stenosis in fourteen patients (70%), vessel rupture in one patient (5%), dissection in one patient (5%), early recurring stenosis and chronic organizing thrombus in one patient (5%) and chronic organizing thrombus and dissection in one patient (5%) (Table 2). 

**Table 2.** Data About Fistula Intervention

	Reason	Approach	Indication
**Case 1**	Occlusion	Venous	COT
**Case 2**	Occlusion	Arterial	RRS
**Case 3**	Occlusion	Venous	RRS
**Case 4**	Occlusion	Venous	RRS + COT
**Case 5**	Stenosis	Venous	Dissection
**Case 6**	Occlusion	Venous	RRS
**Case 7**	Occlusion	Venous	COT
**Case 8**	Stenosis	Venous	RRS
**Case 9**	Occlusion	Arterial	RRS
**Case 10**	Occlusion	Venous	RRS
**Case 11**	Occlusion	Venous	RRS
**Case 12**	Occlusion	Venous	RRS
**Case 13**	Stenosis	Arterial	RRS
**Case 14**	Occlusion	Venous	RRS
**Case 15**	Stenosis	Venous	RRS
**Case 16**	Stenosis	Arterial	Rupture
**Case 17**	Stenosis	Venous	RRS
**Case 18**	Stenosis	Venous	RRS
**Case 19**	Occlusion	Venous	COT + Dissection
**Case 20**	Stenosis	Venous	RRS

Twenty-one stents were used for the twenty patients. Sixteen of them were balloon-expandable (Express stent, Boston Scientific, Natick, Massachusettes, USA; 4-7 mm in diameter, 8-38 mm in length), three were self-expandable nitinol (Protege GPS, Plymouth, MN, USA; 6 mm in diameter, 40-80 mm in length) and one was wall stent(Boston Scientific, Natick, Massachusettes, USA; 10×60 mm in size). One patient had placed two stents in order to obtain the whole length of the stenotic segment (Table 3). 

**Table 3.** Data About Stent Type

	Stent Type	Number of Stents	Size of the Stent (mm)
**Case 1**	Balloon Expandable	1	*4×23*
**Case 2**	Balloon Expandable	1	*7×17*
**Case 3**	Balloon Expandable	1	*7×38*
**Case 4**	Nitinol (Self Expandable)	1	*6×40*
**Case 5**	Balloon Expandable	1	*7×15*
**Case 6**	Balloon Expandable	1	*5×19*
**Case 7**	Balloon Expandable	1	*5×15*
**Case 8**	Balloon Expandable	1	*4×8*
**Case 9**	Balloon Expandable	1	*4×33*
**Case 10**	Balloon Expandable	2	*5×18, 5×14*
**Case 11**	Balloon Expandable	1	*6×20*
**Case 12**	Nitinol (Self Expandable)	1	*6×40*
**Case 13**	Balloon Expandable	1	*4×14*
**Case 14**	Balloon Expandable	1	*5×19*
**Case 15**	Balloon Expandable	1	*4×12*
**Case 16**	Balloon Expandable	1	*7×19*
**Case 17**	Balloon Expandable	1	*6×14*
**Case 18**	Wall Stent	1	*10×60*
**Case 19**	Nitinol (Self Expandable)	1	*6×80*
**Case 20**	Balloon Expandable	1	*6×18*

The clinical success rate was 100%. All the patients were able to achieve successful dialysis treatment. The mean follow-up was 24 months (1-85 months) (Table 4). 

**Table 4.** Follow-Up Data About Fistula Patency

	Follow-Up	Primary Patency Rate	Secondary Patency Rate	Follow-Up Status
**Case 1**	3 months	1 month	3 months	Renal transplantation at 3 months
**Case 2**	4 months	3 months	4 months	4 months patent
**Case 3**	13 months	2 months	13 months	13 months patent
**Case 4**	15 months	6 months	15 months	15 months patent
**Case 5**	7 months	1 month	7 months	7 months patent
**Case 6**	85 months	26 months	85 months	85 months patent
**Case 7**	44 months	12 months	44 months	44 months patent
**Case 8**	2 months	1 month	2 months	2 months Access loss
**Case 9**	18 months	3 months	18 months	18 months patent
**Case 10**	46 months	20 months	46 months	46 months patent
**Case 11**	13 months	1 month	13 months	13 months access loss
**Case 12**	49 months	49 months	49 months	49 months patent
**Case 13**	1 month	1 month	1 month	died 1 month later
**Case 14**	35 months	35 months	35 months	Renal transplantation at 35 months
**Case 15**	46 month	46 months	46 months	4 months patent
**Case 16**	26 month	11 months	26 months	26 months patent
**Case 17**	22 month	9 months	22 months	22 months patent
**Case 18**	15 month	2 months	15 months	15 months patent
**Case 19**	12 month	9 months	12 months	12 months patent
**Case 20**	3 month	3 months	3 months	3 months access loss

**Figure 1.**
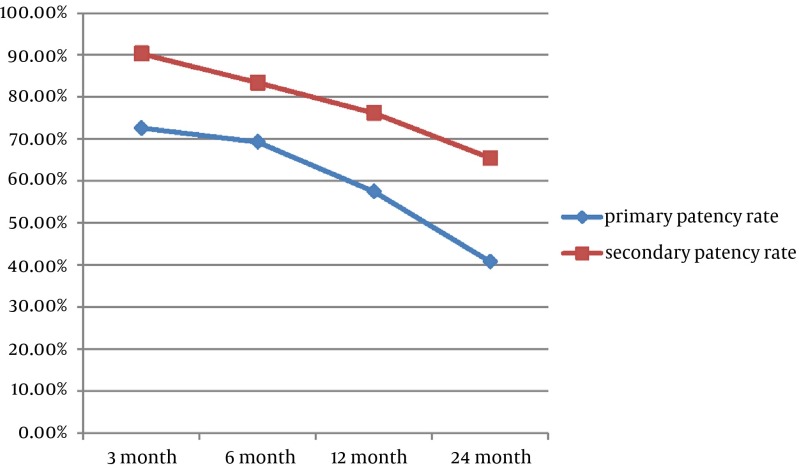
The statistical analyses of the primary and secondary patency rates of the fistulas

**Figure 2.**
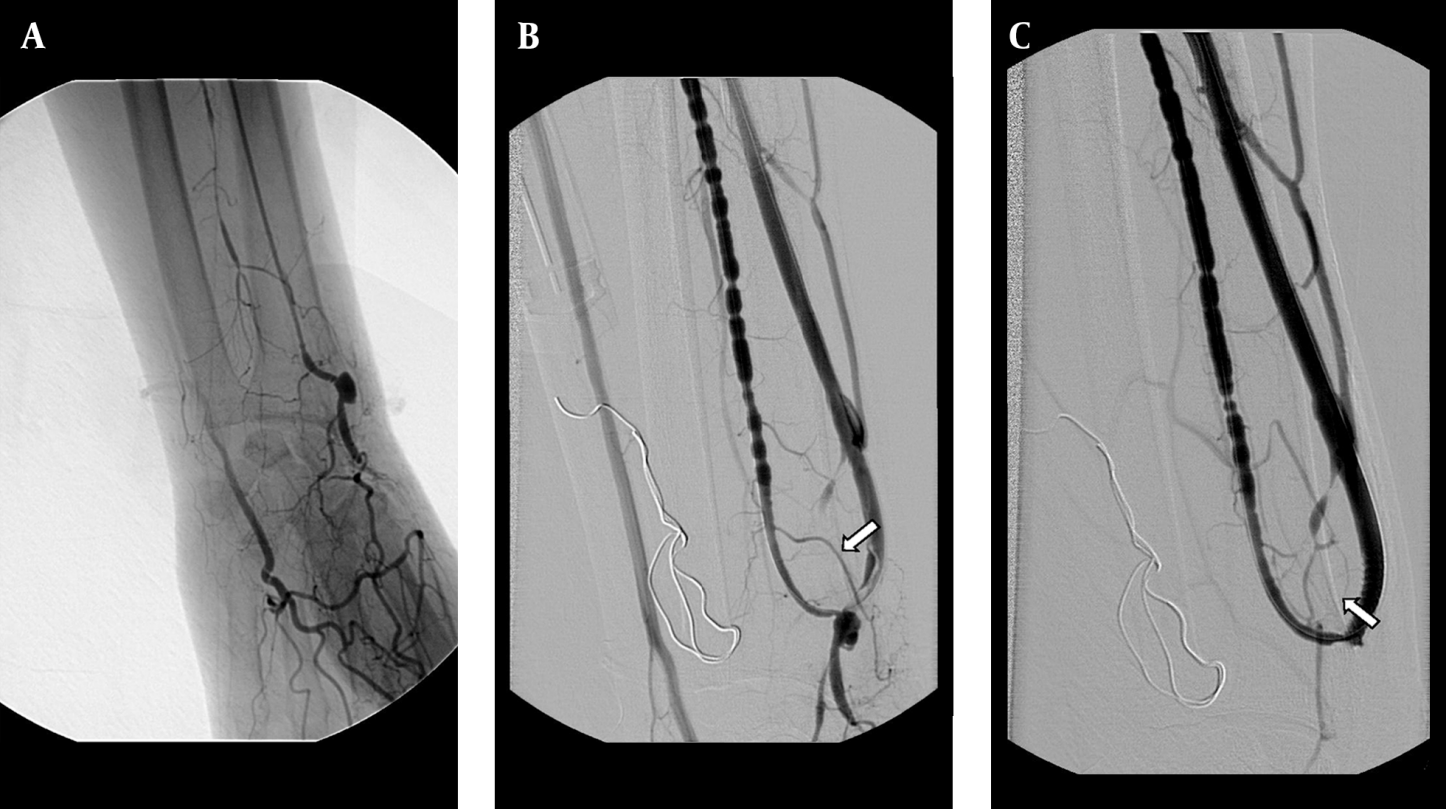
A 48-year-old man with radiocephalic fistula in the left forearm. A, Retrograde catheterization failed to canalize obliterated outflow vein. The fistulogram obtained with the antegrade approach throughout the brachial artery shows the occlusion of the fistula. B, After balloon dilatation (3.5 mm) of the obliterated segment, the dilatation was complicated by dissection at the venous side of the fistula (arrow). C, The 4×23 mm sized stent was placed at the dissected segment and the control fistulogram showed the patency of the fistula and the normal calibration of the venous segment.

The primary patency rates at 3, 6, 12 and 24 months for the AV fistula were 72.6%, 69.3%, 57.5% and 40.8%, respectively. The secondary patency rate was 90.3%, 83.4%, 76.2% and 65.5%, respectively (Figure 1). No major complication occurred throughout the study. Three patients had puncture site hematoma, but no additional interventional procedure was necessary for these minor complications. However, a dissection occurred at the venous site of the AV fistula during the 3.5 mm balloon angioplasty; and a 4 mm diameter and 23 mm length stent was placed at the irregular contour sized venous site. The control fistulogram showed the patency of the AV fistula and the normal calibration of the venous segment (Figure 2). 

During the follow-up procedure, one patient had pseudoaneurysm. In the distal area of the pseudoaneursym, a stenotic segment was seen and a 7×17 mm balloon expandable stent was placed at this site.

**Figure 3.**
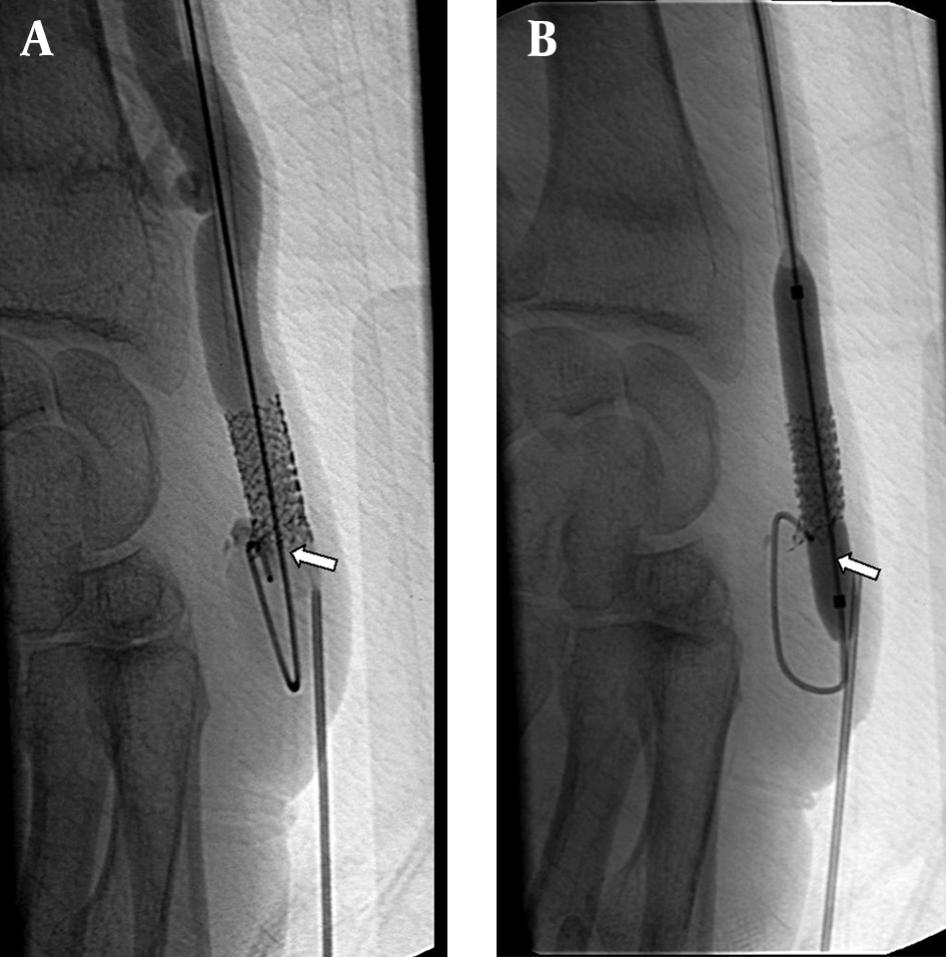
A 53-year-old man with radiocephalic fistula in the left forearm. He had a previous stent placement in this area. A, AV fistulogram shows the thrombus in the stent that occurred in the third month follow-up (arrow). B, The balloon dilatation (6 mm) is used for the stent expandation and a new 7 mm sized second stent is placed at the same area. The control angiogram images show patency of the vascular access (arrow).

**Figure 4.**
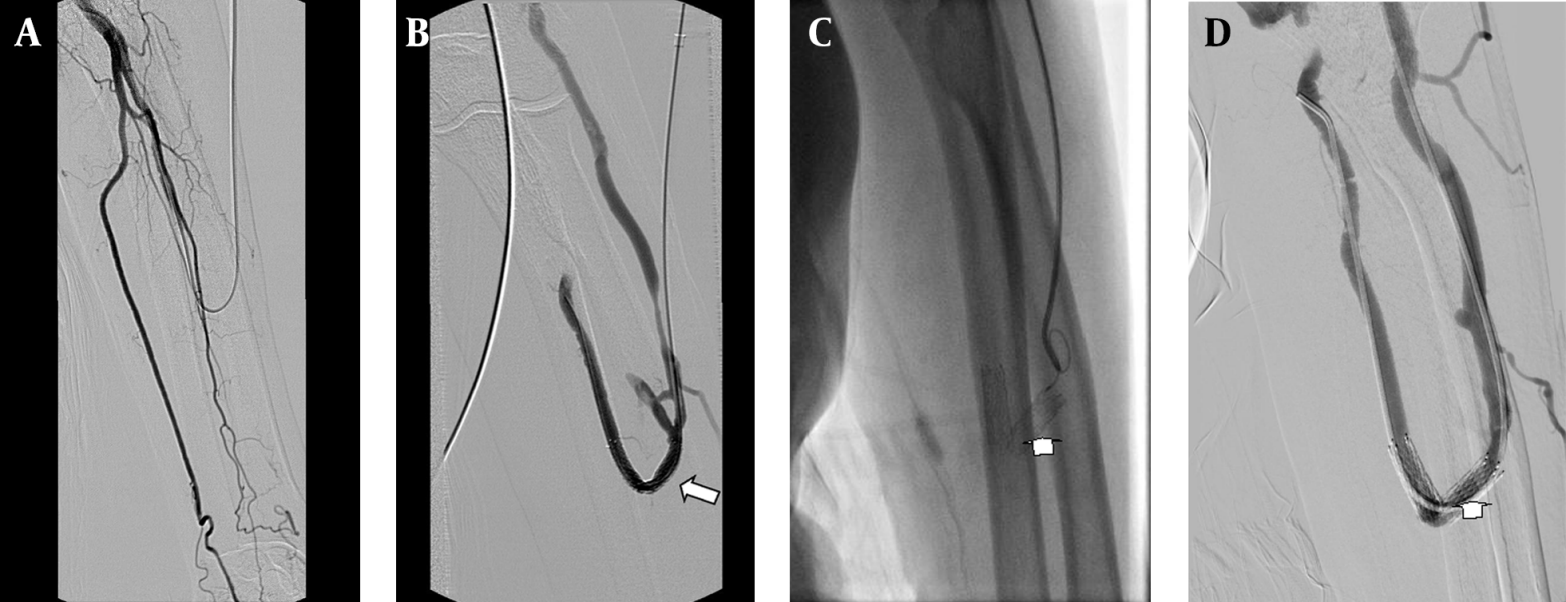
A 75-year-old man with radiocephalic access in the left forearm in place at the third day after PTA; A, Fistulogram shows occlusion of the distal radial artery. B, The metallic stent 6×40 mm deployed at the radial artery and the cephalic vein; the fistulogram shows the blood supply at this access (arrow). C, The follow-up venogram obtained after 8 months with the retrograde catheterization shows laceration of the stent (arrow). D, Angiogram shows patency of the stent and the access (arrow), an additional interventional procedure is not required.

Seventeen additional interventions were performed for 11 (55%) patients due to stent thrombosis or stenosis during the follow-up. In eight patients, one intervention; in one patient, two interventions; in one patient, three interventions and finally in one patient, four interventions were performed (Figure 3). The stent displacement caused inadequate arterial inflow (<200mL/min) in two patients in which the problem was fixed with a second U-shaped stent. The arterial blood inflow was 340 ml/min at follow-up. Stent fracture occurred in one patient at the eight month of follow-up, but the patient’s hemodynamics were good enough for hemodialysis and no additional intervention was needed (Figure 4). In spite of the many additional interventional procedures that were carried out, vascular access dysfunction occurred in three patients at 2,3 and 13 months. Two patients had renal transplantation one of which was accomplished at three months and the other at thirty-five months, although they had patent AV fistula. One patient was dead at the one-month follow-up because of pulmonary infection. 

## 5. Discussion

The most common complication that occurs in hemodialysis patients is the stenosis related with the anastomosis and the drainage veins. Thrombotic complication is the major cause of vascular access loss. The stenosis of AV fistula drainage vein commonly occurs at the AV anastomotic site or at the venous segment, 2-3 cm closer to this area.

The role of metallic stent placement in the peripheral outflow veins in hemodialysis patients is controversial. Most of the articles concerning clinical application of metallic stent in hemodialysis patients reported the treatment of central venous lesions or peripheral lesions in graft patients. Metallic stent usage in peripheral lesions in graft patients has been associated with high success rates (6). Only a few articles described metallic stent placement in native AV fistulas and in only a small number of patients (4). Farber et al. (7) treated peripheral venous lesions with a Dacron (Du-Pont)-covered stent in five AV fistula dialysis patients. The primary and secondary patency rates were 57% and 83%, respectively at 6 months, and 29% and 53%, respectively at the end of the first year. But, the patient population was small, and the indication for stent placement was unclear.

Comparison of the outcomes of surgical versus percutaneous repair of a dysfunctional fistula is confusing. The cumulative patency rates for AV fistulas were not superior with either method. Surgical repair has been reported to achieve cumulative 1-year primary patency rates of approximately 55%. The published report comparing the results of patency rates for arteriovenous fistulas between surgery and PTA as the cost of the charges; endovascular treatment was associated with a higher cost rate than surgical repair (8).

There are several reports that have published promising results of endovascular treatment of stenosed and thrombosed fistulas. In 2000, Turmel-Rodrigues et al. (9) reported primary and secondary 1-year patency rates of 49% and 81%, respectively for failing and thrombosed forearm fistulas. Clark et al. (10) reported a 1-year primary patency rate of 26% and a secondary patency rate of 82% for upper arm and forearm fistulas. In 2004, Rajan et al. (11) published 1-year primary and secondary patency rates of 62% and 86% respectively, for dysfunctional forearm fistulas after attempted endovascular treatment.

Pan et al. (4) treated twelve patients with an AV fistula with metallic stent placement in the peripheral outflow veins to restore vascular access. The indications for metallic stent placement in this study included recoil stenosis of outflow vein in six patients, outflow venous rupture in two patients and dissection and large residual adherent thrombus in outflow aneurysms in three patients with thrombosed access. The primary patency rates of the vascular access at 3, 6, 12 and 24 months was 92%, 81%, 31%, and 31%, respectively. In this study, they mentioned that metallic stent placement is safe and effective in treating peripheral venous lesions in native AV fistula hemodialysis patients after unsatisfactory balloon dilatation. Pan et al. (4) used the transarterial approach to pass the anastomotic stenotic side for the appropriate placement of the stent. In our study, two patients had inadequate arterial inflow due to misplacement of the stent. We used a U-shaped stent for the stenosis at the juxtaanastomotic region.

Indications for stent placement are insufficient balloon angioplasty (residual stenosis greater than 30-50% or stenosis with early recurrence), arterial or venous dissections and ruptures related to angioplasty (4, 6, 7). The indications in our study were the same as in the literature. Moreover, in our study, the indication for stent placement was the chronic organizing thrombus that occurred after insufficient percutaneous thrombolysis. The clinical success rate was 100 % in our study. In other words, all patients had at least one successful dialysis procedure. The primary patency rate in our study was 57.5%, which was higher than the primary patency rate described by the Dialysis Outcomes Quality Initiative guidelines criteria (5). Our one- and two-year patency rates were 76.2% and 65.5%, respectively that are similar to balloon angioplasty and shunt revision rates (12, 13).

The technique that is described in this study has limitations; in the follow-up procedure, more additional interventions are required compared to surgical interventions. The results of the current study are in favor of more long-term surveillance of the access viability.

In conclusion, our study shows that metallic stent placement is a safe and effective procedure for salvage of native hemodialysis fistula after unsuccessful balloon angioplasty in hemodialysis patients. The technical success and access patency rates after stent placement in dialysis patients are considered as satisfactory.
